# E-health literacy is associated with better medication adherence in older adults with chronic comorbidities: the mediating role of information overload and the moderating effect of family care on the anxiety-adherence link

**DOI:** 10.3389/fpubh.2026.1862125

**Published:** 2026-07-10

**Authors:** Rong Hu, Hengyang Wang, Xiangzhi Sun, Lihong Zhang, Sihua Zhao

**Affiliations:** 1 School of Nursing, Lanzhou University, Lanzhou, China; 2 Department of Critical Care Medicine, First Hospital of Lanzhou University, Lanzhou, China; 3 Department of Critical Care Medicine, Chongqing University Cancer Hospital, Chongqing, China; 4 Department of Urology, Ward 2, The First Hospital of Lanzhou University, Lanzhou, China

**Keywords:** anxiety, e-health literacy, information overload, medication adherence, older adults with chronic comorbidities, structural equation modeling

## Abstract

**Objective:**

Using the Stimulus-Organism-Response and stress buffering models, this study explores how e-health literacy is associated with medication adherence in older adults with chronic comorbidities, focusing on the chained mediation of information overload/anxiety and the moderation of family care on anxiety-adherence.

**Method:**

A cross-sectional survey was conducted (October 2025–March 2026) on 428 eligible adults (conveniently sampled from 3 Grade A tertiary hospitals in Northwest China). Assessments used 5 scales (Electronic Health Literacy, Information Overload Severity, Self-Rating Anxiety, Family Care Index, Morisky Medication Adherence). We performed analyses (correlation, stratified regression, structural equation modeling) using SPSS 29.0 and AMOS 26.0.

**Results:**

Medication adherence was poor (80.8% non-adherent), with good model fit. E-health literacy was directly associated with adherence (*β* = 0.234, *p* < 0.001) and indirectly associated via information overload (*β* = 0.022, *p* < 0.05). However, the chained mediation through both information overload and anxiety was not statistically significant; only the single mediation via information overload was significant. Family care moderated the anxiety-adherence association (*β* = 0.102, *p* < 0.001), such that higher care buffered anxiety’s negative impact.

**Conclusion:**

E-health literacy is associated with better adherence in older adults with chronic comorbidities (directly or through reduced information overload), while family care is associated with a weaker negative impact of anxiety on medication behavior. This “moderated mediation” model suggests future interventions may benefit from improving elderly e-health literacy and integrate family care for targeted multi-level strategies.

## Introduction

**Figure 1:**
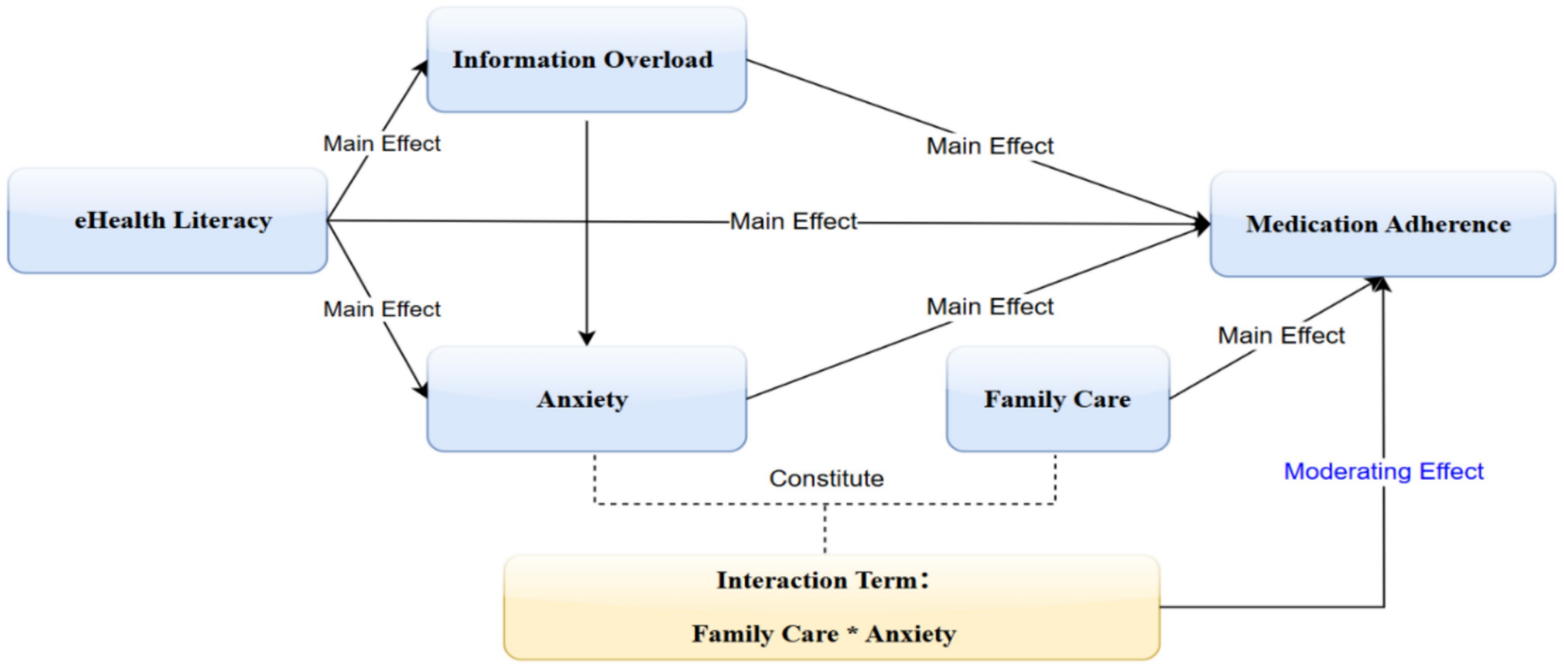
The hypothesized research model based on the S-O-R model. The external stimulus (S) is the digital health information environment (not depicted). e-HL, IO, and ANX are all components of the Organism (O). As a relatively stable capability, e-HL is conceptualized as an antecedent that shapes subsequent cognitive (IO) and emotional (ANX) states within O.

Chronic disease co-morbidity poses a significant health challenge for the older population, and for older adults with chronic comorbidities (OACCs), adherence to complex medication regimens has become even more critical for disease management (1). The internet is currently reshaping how health information is accessed, and e-health literacy (e-HL)—the ability of individuals to locate, understand, and evaluate online health information—has become a vital tool for personal health self-management (2). However, older adults with multiple comorbidities must manage numerous health conditions, and the vast amount of specialized yet potentially conflicting health information available online starkly contrasts with their limited information processing capacity. Although studies have demonstrated that e-HL is associated with healthy behaviors (3), the pathways through which it relates to medication adherence (MA) among OACCs remain to be explored. Existing research has primarily focused on direct relationships between variables, failing to fully elucidate the underlying pathways between e-HL and MA. This study draws on the Stimulus-Organism-Response Model (S-O-R) to construct a chained mediation model that illustrates the proposed pathway. The S-O-R is a psychological model explaining how individuals respond behaviorally to external environmental stimuli through their internal physiological states, and is frequently used to analyze the impact of environmental stimuli on human behavior (4). S-O-R: S corresponds to external environmental stimuli, O corresponds to the organism’s cognitive assessment and emotional response, and R corresponds to behavioral response (5). According to the S-O-R model, e-health literacy is conceptualized as an individual capability that shapes how older adults perceive and process the digital health information environment (the ‘Stimulus’). In the current study, the external stimulus (S) is conceptualized as the complex digital health information environment. Although not directly measured, it serves as the contextual trigger. The Organism (O) includes three interrelated components: e-health literacy (an individual capability), information overload (a cognitive state), and anxiety (an emotional state). As a relatively stable capability, e-HL is conceptualized as an antecedent that shapes subsequent cognitive and emotional states within O. Adults with low e-HL are more prone to experience information overload (IO)—a state of cognitive confusion triggered by excessive health information—which aligns with the “Organism (O)” dimension in the S-O-R model. This cognitive disorientation and associated frustration readily induce negative emotional responses such as anxiety (ANX), another key component of the “O” dimension. Ultimately, these cognitive and emotional disturbances impair both the willingness and capacity of adults to adhere to prescribed medication regimens, leading to reduced MA, which corresponds to the “Response (R)” dimension in the model. However, the connection between psychological states and behavioral outcomes is not inevitable. According to the stress-buffering model (6), social support may help buffer the negative impact of stressful events (anxiety) on an individual’s physical and mental health. Among numerous sources of social support, family care (FC) holds an irreplaceable central position for older adults in China (7). It may serve as a crucial protective resource by providing emotional comfort, simplifying complex information, or directly assisting with medication management. Therefore, we further hypothesize that FC can moderate the path from “O” to “R,” buffering the negative impact of ANX on MA. The theoretical model diagram for this study is shown in Figure 1.

In summary, this study aims to examine a chain mediation model with moderation. Specific research objectives include: (1) validating the chain mediation effect of IO and ANX between e-HL and MA; (2) investigating the moderating role of FC in the relationship between ANX and MA. This study not only contributes to deepening the theoretical understanding of the digital health behaviors but also provides an evidence base for developing precise, multi-level health intervention strategies targeting OACCs and their families.

## Methods

### Research design

This study employed a cross-sectional survey design, selecting older adults with chronic comorbidities who received initial or follow-up outpatient care at three Grade A tertiary hospitals in Northwest China between October 2025 and march 2026 as the study subjects. Inclusion Criteria: (1) Concurrent presence of two or more chronic diseases with confirmed medical diagnosis; (2) Basic literacy and verbal communication skills; (3) Informed consent and voluntary participation; (4) Patient age ≥ 60 years; (5) Confirmed prior use of electronic devices. Exclusion Criteria: (1) Concurrent severe physical illnesses rendering cooperation impossible; (2) History of psychiatric disorders; (3) Use of sedatives or hypnotics; (4) Significant errors in questionnaire completion.

### Measures

*General Information Questionnaire*. Based on literature review and research team discussions, the investigator designed the General Information Questionnaire independently. It includes general demographic factors (age, gender, ethnicity, marital status, education level, occupation, place of residence, housing status, occupation, number of children, monthly personal income), medical information (types of daily medications, disease types, number of comorbidities), and internet usage (length of internet use, average weekly internet usage time).

*e-Health Literacy Scale*. The e-Health Literacy Scale was developed jointly by Norman and Skinner (8). The scale comprises three dimensions with eight items, using a 5-point Likert scale where 1–5 represent “strongly disagree” to “strongly agree.” Total scores range from 8 to 40 points, with <26 indicating low e-health literacy and ≥26 indicating high e-health literacy. Xie et al. (9) validated the psychometric properties of this scale among Chinese older adult populations.

*The Information Overload Severity Scale*. The Information Overload Severity Scale (IOSS) was developed by Yang et al. (10) in 2020. This 7-item scale employs a 5-point Likert scale, with scores ranging from 0 to 4 representing “not at all,” “rarely,” “sometimes,” “often,” and “always,” respectively. The total score ranges from 0 to 28, with higher scores indicating greater severity of information overload. The IOSS items are simple and easy to administer, making it suitable for all age groups in China. Gao et al. validated the psychometric properties of this scale among Chinese older adult populations (11).

*The Morisky Medication Adherence Scale* (23). The Morisky Medication Adherence Scale (12) has demonstrated significant value as a concise and effective assessment tool for evaluating medication adherence among adults with chronic conditions. The scale comprises 8 items. Items 1–7 are scored as follows: “Yes” = 0 points, “No” = 1 point. Item 5 is reverse-scored. Item 8 uses a 5-point Likert scale, scored as 0, 0.25, 0.5, 0.75, and 1 point, respectively. The maximum score is 8 points, with scores below 6 indicating non-adherence and scores of 6 or above indicating adherence. Wang et al. (13) validated the psychometric properties of this scale among the Chinese older adult populations. Permission for the questionnaire (MMAS-8) was formally obtained.

*Self-Rating Anxiety Scale*. The Self-Rating Anxiety Scale comprises 20 items scored on a 1–4 scale. Items 5, 9, 13, 17, and 19 are reverse-scored, with scores ranging from 4 to 1. Sum the scores of all 20 items to obtain the raw score. Multiply the raw score by 1.25 and round to the nearest whole number to derive the standardized score. Based on Chinese normative data, the SAS cutoff score is 50 points: - 50–59 points: Mild anxiety - 60–69 points: Moderate anxiety - 70 points and above: Severe anxiety In recent years, the SAS has been adopted as a self-report tool for assessing anxiety symptoms in counseling clinics, demonstrating good reliability and validity (13, 14).

*Family Adaptation, Partnership, Growth, Affection, and Resolve Scale*. Family Adaptation, Partnership, Growth, Affection, and Resolve scale is an assessment tool developed by American scholar Smilkstein to evaluate individuals’ satisfaction with their family functioning (15). This 5-item scale employs a 3-point Likert scoring system, yielding a total score ranging from 0 to 10. Higher scores indicate better family care. A score of 0–3 indicates poor family care, 4–6 represents average family care, and 7–10 indicates good family care. Widely used in China, this scale has demonstrated sound psychometric properties among the Chinese elderly population (16).

### Statistical analysis

Data entry and organization were performed using Excel, while statistical analysis was conducted with SPSS 29.0 and AMOS 26.0. Two-tailed tests were employed, with *p* < 0.05 indicating statistically significant differences.

(1) Descriptive analysis: categorical variables were described using N (%). Continuous variables meeting normal distribution were described using (x ± s); those not meeting this criterion were described using M (q1, q3).
(2) Correlation analysis: Pearson correlation analysis was employed to examine the relationships among core variables including e-HL, IO, ANX, FC, and MA.
(3) Stratified regression analysis: to assess the independent predictive role of core variables on MA while controlling for demographic and clinical factors, stratified regression analysis was conducted. The first layer (Model 1) included all control variables (age, gender, etc.). The second layer (Model 2) incorporated core variables—e-HL, IO, ANX, FC—on top of Model 1. Incremental explanatory variance of core variables was assessed by comparing *ΔR^2^*. To assess common method bias, Harman’s single-factor test was conducted using all items of the five scales. The first unrotated factor explained 31.02% of the total variance, below the 50% threshold, indicating that common method bias is not a serious concern.
(4) Structural equation modeling analysis: to examine the mediating and moderating effects within the theoretical model, a structural equation model was constructed using AMOS 26.0. Since the data were approximately normally distributed, maximum likelihood estimation was employed. The chained mediating effect of IO on ANX was tested using Bootstrap sampling (5,000 repetitions). A significant mediating effect was indicated if the 95% confidence interval did not include zero. To examine the moderating effect, ANX and FC were centered, and their interaction term was included in the model. A significant path coefficient for the interaction term confirmed the moderating effect. The analysis was a path analysis using observed total scores, not a latent-variable SEM. Control variables were excluded from this SEM model to maintain the theoretical model’s simplicity, as the hierarchical regression (Table 1) had already demonstrated that covariates explained limited incremental variance (ΔR^2^ = 0.120, *p* < 0.001) after controlling for all covariates.”

**Table 1:** Results of hierarchical regression analysis for medication adherence (*n* = 428).

Characteristics	Model 1			Model 2		
	*β*	*t*	*P*	*β*	*t*	*P*
No. of internet devices	−0.121	−2.463	**0.014**	−0.130	−2.850	**0.005**
No. of daily meds types	−0.444	−6.702	**<0.001**	−0.408	−6.598	**<0.001**
Educational level	0.195	3.780	**<0.001**	0.133	2.735	**0.007**
Other control variables	/	/	>0.05	/	/	>0.05
IO				−0.135	−3.112	**0.002**
e-HL				0.216	4.356	**<0.001**
ANX				−0.188	−4.371	**<0.001**
FC				0.166	3.826	**<0.001**
*R^2^*	0.183			0.303		
*ΔR^2^*	0.120					
*F*	4.807^**^			7.635^**^		

IO, Information Overload; e-HL, eHealth Literacy; ANX, Anxiety; FC, Family Care; Bold text indicates *P* < 0.05.

In summary, based on the S-O-R model, stress buffering model, and research objectives, we constructed the path diagram for the structural equation model of this study (see Figure 1). As shown, the direct effects in this model are: ① e-HL → IO; ② e-HL → ANX; ③ e-HL → MA; ④ IO → MA; ⑤ IO → ANX; ⑥ ANX → MA; ⑦ FC → MA. Indirect effects: ① e-HL → IO → ANX → MA; ② e-HL → IO → MA; ③ e-HL → ANX → MA. Moderating effect: (ANX × FC) → MA.

## Results

### Participant characteristics

A total of 455 questionnaires were distributed, with 428 valid responses collected. The subjects ranged in age from 60 to 90 years, with a mean age of (67.2 ± 6.4) years. Among all subjects, males accounted for 48.4% and females for 51.6%; 55.1% of subjects were from rural areas; and 87.9% had a personal monthly income below 5,000 yuan. Further details are provided in Table 2.

**Table 2:** Background characteristics of participants (*N* = 428).

Characteristics	M ± SD/*N* (%)
Ages (years)	67.2 ± 6.4
Internet usage tenure (years)	3.11 ± 1.1
Weekly internet usage duration	4.5 ± 3.4
No. of internet devices	1.8 ± 0.7
No. of children	2.5 ± 0.7
No. of chronic diseases	2.7 ± 0.6
No. of daily meds types	3.9 ± 1.4
Gender	
Male	207 (48.4)
Female	221 (51.6)
Ethnic	
Han nationality	334 (78.0)
Minority	94 (22.0)
Geographical location	
Town	192 (44.9)
Countryside	236 (55.1)
Cohabitation situation	
Living together as a couple	223 (52.3)
Living alone	151 (35.3)
Living with children	54 (12.6)
Educational level	
Elementary school and below	127 (29.7)
Secondary school	151 (35.3)
Junior college	116 (27.1)
Undergraduate and above	34 (7.9)
Occupation	
Farmers or workers	193 (45.1)
Public-sector or medical professionals	74 (17.3)
Freelance occupation	161 (37.6)
Marital status	
Married	314 (73.4)
Non-married (widowed, divorced/separated)	114 (26.6)
Personal physical health status	
Poor	81 (18.5)
Fair	264 (61.7)
Good	83 (19.4)
Personal monthly income (RMB)	
<2000	133 (31.1)
2000~5,000	243 (56.8)
>5,000	52 (12.1)

### Profiles of and correlations between measures

A survey revealed that the overall MA score among 428 OACCs was (4.29 ± 1.27). Among them, 346 older adults (80.8%) scored below 6 points (indicating low adherence), while 82 older adults (19.2%) scored 6 points or higher (indicating high adherence). These findings indicate that MA is generally poor among this patient population. Pearson correlation analysis revealed that MA was negatively correlated with IO (*r* = −0.226, *p* < 0.001) and ANX (*r* = −0.191, *p* < 0.001), positively correlated with e-HL (*r* = 0.261, *p* < 0.001), and showed a significant positive correlation (*r* = 0.222, *p* < 0.001) with FC. See Table 3 for details.

**Table 3:** Profiles of measures and the correlations between them.

	SR	Mean ± SD	1	2	3	4
MA	0 ~ 8	4.29 ± 1.27				
Correlation, r			/			
*P*			/			
IO	0 ~ 28	13.57 ± 7.20				
Correlation, r			−0.226	/		
*P*			**<0.001**	/		
e-HL	8 ~ 40	20.85 ± 7.78				
Correlation, r			0.261	−0.125	/	
*P*			**<0.001**	**0.010**	/	
ANX	25 ~ 100	63.20 ± 20.93				
Correlation, r			−0.191	0.046	−0.011	/
*P*			**<0.001**	0.347	0.828	/
FC	0 ~ 10	6.25 ± 2.23				
Correlation, r			0.222	−0.116	0.052	0.041
*P*			**<0.001**	**0.017**	0.279	0.403

1 = MA: Medication Adherence; 2 = IO: Information Overload; 3 = e-HL: e-Health Literacy; 4 = ANX: Anxiety; 5 = FC: Family Care; Bold text indicates *p* < 0.05.

### Hierarchical regression

To examine the independent predictive effects of demographic factors, clinical factors, and core psychological variables on MA, we conducted hierarchical regression analyses, with the results presented in Table 1. Model 1 incorporated control variables (age, gender, educational attainment, monthly personal income, number of comorbidities, types of daily medications, etc.) into the equation. Results indicated that these variables collectively explained 18.3% of the variance in MA (*F* = 4.807, *p* < 0.001). Model 2 incorporated core variables (e-HL, IO, etc.) alongside those in Model 1. Results indicated that including these core variables significantly improved the model’s explanatory power for MA, with *ΔR^2^* = 0.120. After controlling for demographic and clinical factors, e-HL, IO, ANX, and FC emerged as significant predictors of MA. Ultimately, all variables collectively explained 30.3% of the variance in MA (*F* = 7.635, *p* < 0.001).

### Structural equation modeling

#### Model fit

This study employed maximum likelihood estimation to fit the theoretical model. The model fit indices are as follows: χ^2^ = 10.1, df = 5, χ^2^/df = 2.020, SRMR = 0.037, RMSEA = 0.049, GFI = 0.992, CFI = 0.947, NFI = 0.910, IFI = 0.952, TLI = 0.842. Although the TLI value is slightly below the conventional standard of 0.90, the other fit indices (CFI = 0.947, RMSEA = 0.049, SRMR = 0.037) all indicate acceptable fit. Therefore, we consider the model acceptable while acknowledging this limitation.

#### Path coefficient analysis

The model results indicate that among the direct pathways, neither the effect of e-HL on ANX nor the effect of IO on ANX reached statistical significance (*β* = −0.005, *p* = 0.919; *β* = 0.045, *p* = 0.356), while all other direct pathways were statistically significant. Additionally, the interaction term between ANX and FC exerted a significant positive effect on MA (*β* = 0.102, *p* < 0.001), indicating that FC moderates the relationship between ANX and MA. Detailed results are presented in Table 4.

**Table 4:** Standardized path coefficients of the structural equation model.

Path relationship	*β*	*SE*	*CR*	*P*
e-HL → IO	−0.125	0.044	2.593	**0.010**
e-HL → MA	0.234	0.007	4.639	**<0.001**
e-HL → ANX	−0.005	0.131	−0.101	0.919
IO→MA	−0.172	0.007	−3.29	**<0.001**
IO→ANX	0.045	0.142	0.923	0.356
ANX → MA	−0.189	0.002	−4.613	**<0.001**
FC → MA	0.209	0.023	4.454	**<0.001**
ANX*FC → MA	0.102	0.007	3.229	**<0.001**

1 = MA, Medication Adherence; 2 = IO, Information Overload; 3 = e-HL, eHealth Literacy; 4 = ANX, Anxiety; 5 = FC, Family Care; β, Standardized Coefficient; Bold text indicates *P* < 0.05.

#### Direct effect, indirect effect, and total effect

Further examining the mediating effect and total effect, the results are shown in Table 5. The total effect of e-HL on MA was 0.256 (*p* < 0.001), comprising a direct effect of 0.234 and an indirect effect of 0.022. MA was significant (*β* = 0.022, *p* < 0.05), whereas the chain mediating pathway from IO → ANX → MA did not reach statistical significance (*β* = 0.001, *p* > 0.05).

**Table 5:** Analysis of direct, indirect, and total effects.

Path relationship	Direct effect	Indirect effect	Total effect	95%CI
e-HL → IO	−0.125		**−0.125** ^*^	[−0.204, −0.025]
e-HL → MA	0.234	0.022	**0.256** ^**^	[0.023, 0.052]
e-HL → ANX	−0.005		−0.005	[−0.290, 0.258]
IO → MA	−0.172	−0.009	**−0.181** ^**^	[−0.045, −0.016]
IO → ANX	0.045		0.045	[−0.137, 0.406]
ANX → MA	−0.189		**−0.189** ^*^	[−0.017, −0.007]
FC → MA	0.209		**0.209** ^**^	[0.070, 0.169]
e-HL → IO → ANX → MA		(−0.125) * (0.045) * (−0.189)	0.001	[0.000, 0.001]
e-HL → IO → MA		(−0.125) * (−0.172)	**0.022** ^*^	[0.001, 0.008]
e-HL → ANX → MA		(−0.005) * (−0.189)	0.000	[−0.003, 0.003]
Moderating effect				
ANX * FC → MA	0.102		**0.102** ^**^	[0.014, 0.179]

*p < 0.05; ^**^p < 0.01; MA, Medication Adherence; IO, Information Overload; e-HL, e-Health Literacy; ANX, Anxiety; FC, Family Care.

#### Moderating effect analysis

**Figure 2:**
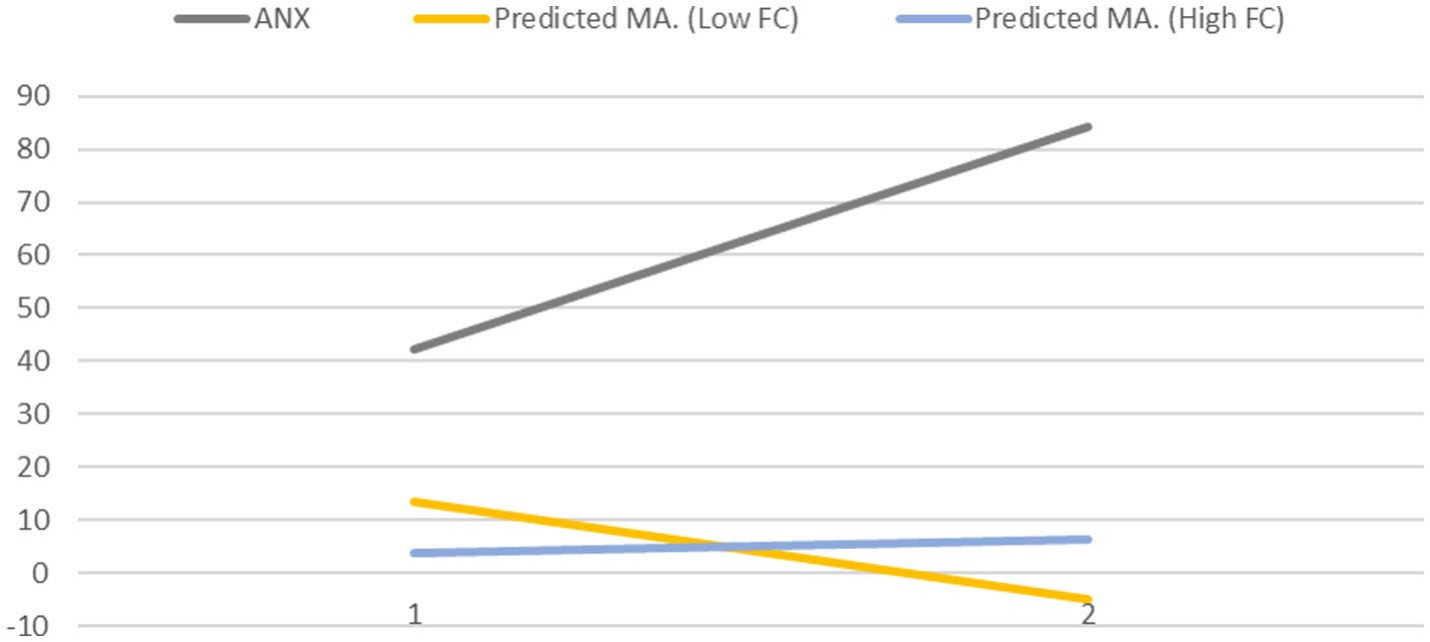
The relationship between anxiety and medication adherence at different levels of family care. Predicted MA, (Low FC), Predicted Medication Adherence (Low Family care); Predicted MA, (High FC), Predicted Medication Adherence (High Family Care); ANX, Anxiety.

To examine the moderating role of FC between ANX and MA, we centered the ANX and FC variables and included their interaction term in the model. Results indicated that the path coefficient of this interaction term was significantly positive (*β* = 0.102, *p* < 0.001). This positive coefficient indicates that higher levels of FC mitigate the negative impact of ANX on MA, acting as a buffer. Further simple slope analysis (see Figure 2) clearly illustrates this pattern: at low levels of FC, ANX exerts a stronger negative influence on MA (steeper slope), whereas at high levels of FC, this negative effect is significantly attenuated (slope becomes flatter).

## Discussion

Survey results indicate that among 428 OACCs, only 19.2% demonstrated good MA, while a high proportion of 80.8% exhibited poor MA. This indicates that MA among OACCs is generally low. This finding significantly exceeds previous scholarly conclusions that “the proportion of older adults with chronic conditions with poor MA ranges from 40 to 75% (17). The primary reasons for this phenomenon may be explained by three key factors: First, OACCs typically suffer from a greater variety of diseases compared to those with common chronic conditions, which may be associated with a significant increase in the number and types of medications they take. Second, memory decline is prevalent among the elderly population. Combined with the fact that some drug names are lengthy and difficult to remember, this may often be related to missed doses. Third, some OACCs may choose to reduce their dosage or discontinue medication on their own when symptoms are not pronounced, which may be associated with further reductions in MA.

The results of the hierarchical regression analysis in this study indicate that after controlling for demographic and clinical factors (e.g., age, types of daily medications), the inclusion of core variables (e-HL, IO, etc.) significantly increased the model’s explanatory power for MA by 12.0%. This finding suggests that the digital health psychological variables examined in this study possess unique and important explanatory power for MA among OACCs. Hierarchical regression analysis identified several key factors influencing MA. Among these, the number of daily medications and the number of internet-connected devices may increase the management burden for OACCs from the dimensions of clinical complexity and information resource accessibility, thereby posing challenges to MA. Furthermore, as a fundamental personal competency, educational attainment has once again been found to be positively associated with adherence (18), suggesting that a higher level of education may be related to better adherence behaviors, possibly through enhanced information comprehension and management capabilities.

The results of the correlation analysis indicate that e-HL is negatively correlated with IO (*r* = −0.125, *p* = 0.010) and positively correlated with MA (*r* = 0.261, *p* < 0.001); IO is negatively correlated with MA (*r* = −0.226, *p* < 0.001). Further path analysis results indicate that e-HL not only exerts a significant direct positive effect on MA (*β* = 0.234, *p* < 0.001), but also exerts an indirect effect mediated by IO (*β* = 0.022, *p* < 0.05). This result supports that e-HL, as a personal capacity resource, is a direct facilitator of health behaviors (e.g., regular medication use), which is consistent with previous research findings (2, 19). Although this indirect effect was statistically significant, its magnitude (*β* = 0.022) was modest, suggesting that the practical impact of this pathway may be limited. This small effect size indicates that while the mediating role of information overload is statistically detectable, its clinical or practical relevance may be minimal, and the pathway should be interpreted with caution. The mediating pathway “e-HL → IO → MA” is significant, which is consistent with the S-O-R model (20). Specifically, low e-HL (an individual trait) makes OACCs more susceptible to IO (a state of cognitive confusion that represents the cognitive evaluation in the “Organism” component) when they are exposed to complex digital health information environments (the “Stimulus” component). This cognitive overload may impair their ability to maintain consistent MA behaviors (the “Response” component). However, in this study, the theoretical hypotheses—specifically the “e-HL → ANX” and “IO → ANX” pathways, as well as the complete chain mediating pathway “e-HL → IO → ANX → MA”—failed to reach statistical significance. Possible reasons include the following: First, for OACCs, MA may be more habit-driven and task-oriented. Cognitive confusion (IO) may more directly reflect a disruption of their specific ability to perform medication-related tasks (e.g., forgetting complex dosing instructions) rather than necessarily triggering generalized ANX first. In other words, the pathway from “cognitive appraisal” to “behavioral response” may be more direct and impactful than the pathway mediated by “emotional response.” Second, this study measured generalized anxiety using the Self-Rating Anxiety Scale. However, the ANX experienced by older adults may stem more from concerns about the disease itself, medical costs, or functional decline, rather than primarily from health information processing (21). This may explain why the correlations between e-HL, IO, and the ANX measured in this study are not strong.

To summarize the empirical support for our hypothesized model: (1) Supported pathways include the direct association between e-HL and MA, the indirect association via IO (e-HL → IO → MA), and the moderating role of FC on the anxiety–MA relationship. (2) Non-supported pathways include the direct paths from e-HL to ANX and from IO to ANX, as well as the full chained mediation (e-HL → IO → ANX → MA). Thus, the S-O-R model is partially consistent with the observed associations in this sample of older adults with chronic comorbidities. However, given the cross-sectional design, these findings should be interpreted as associations consistent with the model rather than as evidence of causal pathways.

The findings of this study reveal that FC exerts a significant moderating effect on the relationship between ANX and MA (*β* = 0.102, *p* < 0.001). To our knowledge, this study provides preliminary evidence—within the context of digital health and specifically among OACCs—that FC can mitigate the negative impact of ANX on MA. This finding provides new empirical support and practical implications for the “Stress Buffering Model.” This model posits that social support mitigates the detrimental effects of stress on health outcomes (22). Specifically, this study suggests that within complex health information environments, FC—as a crucial form of social support—effectively alleviates the corrosive impact of ANX on MA. More importantly, this study indicates through SEM that the buffering effect of FC operates along the pathway from “emotional response” to “final behavior”—i.e., the “O → R” pathway in the S-O-R model. This positioning deepens our understanding of the process underlying social support: when OACCs experience ANX due to cognitive overload (IO) or other reasons amid overwhelming digital health information, high levels of FC may help stabilize their emotions through emotional comfort and reduce behavioral barriers via instrumental support (e.g., assisting with medication organization and reminding older adults to take medications). Thus, this finding of the present study may expand the explanatory boundaries of both the Stress Buffering Model and the S-O-R model. It suggests that to promote the health of elderly individuals in the digital era, we must not only focus on individuals’ information processing capabilities (i.e., e-HL) but also recognize the family system as a critical protective factor that requires targeted intervention. It also clarifies the precise position of FC within the digital health S-O-R chain of e-HL → cognitive appraisal → health behaviors.

### Limitation

(1) Design and Sample: The cross-sectional design limits the establishment of causality; the sample was restricted to three tertiary hospitals using convenience sampling, and only included older adults with prior experience using electronic devices (Inclusion Criterion 5). This inclusion criterion may introduce selection bias, as older adults without digital access or skills were systematically excluded. Therefore, the findings may not be generalizable to the broader population of older adults with chronic comorbidities, particularly those without digital access or skills.
(2) Variable Measurement: Core variables rely on self-reporting, exhibiting significant subjective bias and lacking objective validation; partial scale adaptability issues and incomplete definitions of key variables. Additionally, self-reported measures may be subject to social desirability bias, as participants may have over-reported adherence or family care to present themselves favorably.
(3) Structural equation modeling, while focused on examining theoretical pathways, may overlook significant control variables not included in hierarchical regression. This omission could lead to biased interpretations of the overall mechanism, particularly regarding indirect paths such as “control variables → mediating variables → compliance.”
(4) Although Harman’s single-factor test suggested that common method bias was not severe, all core variables were measured using self-report questionnaires at a single time point. This may still introduce shared method variance. Future studies should consider using objective measures or longitudinal designs.

## Data Availability

The raw data supporting the conclusions of this article will be made available by the authors, without undue reservation.
